# Risk factors for poor outcome after aneurysmal subarachnoid hemorrhage in patients with initial favorable neurological status

**DOI:** 10.1007/s00701-024-05968-5

**Published:** 2024-02-20

**Authors:** Annika Lenkeit, Marvin Darkwah Oppong, Thiemo Florin Dinger, Meltem Gümüs, Laurèl Rauschenbach, Mehdi Chihi, Yahya Ahmadipour, Anne-Kathrin Uerschels, Philipp Dammann, Cornelius Deuschl, Karsten H. Wrede, Ulrich Sure, Ramazan Jabbarli

**Affiliations:** 1 Department of Neurosurgery and Spine Surgery, University Hospital Essen, Hufelandstrasse 55, 45147 Essen, Germany; 2 Department of Diagnostic and Interventional Radiology and Neuroradiology, University Hospital Essen, Essen, Germany

**Keywords:** Subarachnoid hemorrhage, Favorable-grade SAH, Outcome predictors, Functional outcome

## Abstract

**Background:**

Aneurysmal subarachnoid hemorrhage (aSAH) remains a devastating diagnosis. A poor outcome is known to be highly dependent on the initial neurological status. Our goal was to identify other parameters that favor the risk of complications and poor outcome in patients with aSAH and initially favorable neurologic status.

**Methods:**

Consecutive aSAH cases treated at our hospital between 01/2003 and 06/2016 with the initial World Federation of Neurosurgical Societies grades I–III were included. Data on demographic characteristics, previous medical history, initial aSAH severity, and functional outcome after aSAH were collected. The study endpoints were the occurrence of cerebral infarcts, in-hospital mortality, and unfavorable outcome at 6 months after aSAH (modified Rankin scale > 3).

**Results:**

In the final cohort (*n*= 582), the rate of cerebral infarction, in-hospital mortality, and unfavorable outcome was 35.1%, 8.1%, and 17.6% respectively. The risk of cerebral infarction was independently related to the presence of acute hydrocephalus (adjusted odds ratio [aOR]=2.33, *p*<0.0001), aneurysm clipping (aOR=1.78, *p*=0.003), and use of calcium channel blockers concomitant to nimodipine (aOR=2.63, *p*=0.002). Patients’ age (>55 years, aOR=4.24, *p*<0.0001), acute hydrocephalus (aOR=2.43, *p*=0.036), and clipping (aOR=2.86,* p*=0.001) predicted in-hospital mortality. Baseline characteristics associated with unfavorable outcome at 6 months were age (aOR=2.77, *p*=<0.0001), Fisher grades III–IV (aOR=2.81, *p*=0.016), acute hydrocephalus (aOR=2.22, *p*=0.012), clipping (aOR=3.98, p<0.0001), admission C-reactive protein>1mg/dL (aOR=1.76, *p*=0.035), and treatment intervals (aOR=0.64 per-5-year-intervals, *p*=0.006).

**Conclusions:**

Although cerebral infarction is a common complication in aSAH individuals with favorable initial clinical condition, >80% of these patients show favorable long-term outcome. The knowledge of outcome-relevant baseline characteristics might help to reduce the burden of further complications and poor outcome in aSAH patients who tolerated the initial bleeding event well.

**Supplementary information:**

The online version contains supplementary material available at 10.1007/s00701-024-05968-5.

## Introduction

The outcome of patients with aneurysmal subarachnoid hemorrhage (aSAH) is poor, as it continues to be a life-threatening type of stroke with high morbidity and mortality [21]. Various baseline factors and early and secondary complications contribute to this. At the onset of disease, initial severity of aSAH and aneurysm re-bleeding are the two main factors impacting further prognosis [5, 20, 28, 32]. There are some clinical and radiological scores for classifying the initial severity of SAH. In particular, the clinical classification scales such as Hunt and Hess [17] or WFNS (World Federation of Neurosurgical Societies) [31] scales have shown good correlation with outcome of aSAH patients in many studies [2, 12, 18, 32]. These clinical scales were reported to reflect the burden of early brain injury (EBI) [30] after aneurysm rupture, which is strongly linked with further course and outcome of disease. Moreover, radiographic characteristics of SAH including the Fisher score [10, 12, 32] presence of intracerebral (ICH) [6, 13] and intraventricular hemorrhage (IVH) [19, 29] were also confirmed as robust predictors of aSAH outcome.

Although the patients with lower WFNS grade have substantially better prognosis than those with high grade, aSAH patients presenting with WFNS grades I–III may also suffer poor outcome despite lower incidence of EBI and maximal treatment [34]. In these patients, the impact of secondary complications such as symptomatic vasospasm [11], delayed cerebral ischemia (DCI), and infections [7, 35] might be of particular importance. Moreover, patients’ age is an acknowledged outcome predictor in this aSAH subpopulation [4, 15]. However, it remains unclear, whether the other baseline characteristics of aSAH patients, particularly their previous medical history, are also of relevance for the further course of aSAH in individuals who overcome the initial bleeding event without severe neurological deterioration. The knowledge of outcome-relevant baseline risk factors might help to prevent secondary neurological complications and poor outcome in these aSAH individuals.

Thus, we aimed to identify the rates of and the risk factors for cerebral infarction, in-hospital mortality, unfavorable 6-month outcome, and occurrence of outcome-relevant secondary complications in aSAH patients with initial favorable clinical condition in a large retrospective monocentric observational cohort study.

## Materials and methods

### Patient population

The study is based on the institutional retrospective database including all consecutive cases with acute aSAH presenting with a WFNS grades I–III at admission, who were treated in our clinic between 01/2003 and 06/2016. Individuals without aneurysm treatment were excluded from the final cohort. The study was approved by the institutional ethics committee (Faculty of Medicine of the University Duisburg-Essen, Registration number: 15-6331-BO) and registered in the German clinical trial register (DRKS, Unique identifier: DRKS00008749).

### SAH management

Clinical management of aSAH in the intensive care unit included neurologic monitoring and blood pressure control. Diagnosis of aSAH was made by a computed tomography (CT) scan and, if negative, by additional lumbar puncture. Digital subtraction angiography (DSA) was performed to identify the ruptured aneurysm. The treatment decision of clipping vs. coiling was determined on an interdisciplinary basis. Acute hydrocephalus was treated by placement of an external ventricular drain (EVD) allowing the monitoring of intracranial pressure (ICP). During postoperative/postinterventional intensive care, transcranial Doppler ultrasound (TCD) was performed at least once daily for 14 days and oral nimodipine (360 mg/day) was taken for 21 days after the onset of aSAH. Mean arterial pressure was raised to >70 mmHg, with further increase to >90 mmHg in individuals suspected for cerebral vasospasm. If refractory symptomatic vasospasm occurred, repeat DSA with intra-arterial spasmolysis was performed. Repeated CT scans were performed during the first 24 h after aneurysm treatment, in cases of neurological worsening, and during EVD weaning.

### Data management

All variables of interest were gathered from the institutional retrospective aSAH database with additional screening of the electronic medical records and the review of the imaging data for radiographic parameters by the senior author (R.J.). The recorded baseline variables included the demographic data, previous medical history (comorbidities and regular medication), parameters of initial clinical (WFNS grade) and radiographic (Fisher grade) severity of aSAH, aneurysm characteristics (size and location of aneurysm, presence of irregularity/daughter sack), and treatment modality (clipping/coiling), as well as the laboratory parameters (white blood cells [WBC] count, hemoglobin, c-reactive protein [CRP], and troponin), blood pressure, and body temperature at admission. The following complications during aSAH have also been recorded: cerebral vasospasm requiring intra-arterial spasmolysis, sustained increase of ICP >20 mmHg requiring conservative/surgical (craniectomy) treatment and occurrence of systemic infections during the initial hospital stay. Finally, the outcome parameters collected for the study included the modified Rankin Scale (mRS) [33] at discharge and 6-month routine outpatient follow-up and occurrence of new cerebral infarctions in the follow-up CT scans up to 6 weeks after SAH.

### Study endpoints and statistical analysis

The main goal of this study was the evaluation of a possible deterioration, morbidity, and mortality in initially favorable-graded aSAH patients defined as WFNS=I–III. The primary study endpoints were the identification of risk factors for the occurrence of cerebral infarctions, in-hospital mortality, and unfavorable outcome at 6 months defined as mRS>3. As secondary study endpoints, the risk factors for cerebral vasospasm treated with intra-arterial spasmolysis, ICP increase requiring treatment, and systemic infection were analyzed.

Possible associations were first evaluated in univariate analysis using the Student’s *t*-test for normally distributed and Mann-Whitney* U*-test for non-normally distributed continuous data, and Fisher’s exact or chi-square test for dichotomous and categorical variables. Significant results were then included in a multivariable binary logistic regression analysis. For the final tests, the continuous and categorical variables were dichotomized according to the cohorts’ median value (age at 55 years), common cutoffs (Fisher scale as low and high grades, III–IV vs I–II respectively) or upon the receiver operating characteristic curve analysis (laboratory values). Additionally, the effect of treatment updates in SAH management [3] over the observation period 2003–2016 on the study endpoints was also analyzed by splitting the cohort in 5-year intervals. The multivariable regression analyses were also adjusted for time periods.

Statistical analysis was performed using IBM SPSS Statistics version 26 (SPSS Inc., IBM Corp., North Castle, New York, USA). Differences with a *p*-value < 0.05 were considered statistically significant.

## Results

### Population characteristics

Between 01/2003 and 06/2016, 994 patients with acute aneurysmal SAH were treated in our institution. Of them, 582 subjects who had WFNS grades I–III at the time of admission and underwent aneurysm treatment were included in our study. A total of 394 (67.6%) were females and 188 (32.4%) males, median age was 55 years (range 13–90 years). Microsurgical clipping was performed in 203 (35.6%) patients, with no relevant change in the ratio between clipping and coiling between 2003 and 2016 (*p*=0.307). A detailed description of the cohort characteristics is given in Table 1.

**Table 1:** Descriptive analysis of the cohort characteristics

Parameter	Count/mean	Percentage*/SD
Demographic parameters		
Age>55 years	231	39.7%
Sex (female)	394	67.7%
Previous medical history		
Arterial hypertension	397	68.4%
Hypothyroidism	64	11.0%
Hyperthyroidism	5	0.9%
Hyperuricemia	17	2.9%
Diabetes mellitus	29	5.0%
Statin	28	4.9%
Anticoagulants	44	7.6%
NSAID	46	8.0%
Beta blockers	82	14.4%
Calcium channel blockers	55	9.6%
ACE inhibitors	87	15.3%
AT1 receptor blockers	30	5.3%
SAH characteristics		
Fisher scale, grades III–IV	356	75.3%
Acute hydrocephalus	332	57.0%
Aneurysm location		
Middle cerebral artery	128	22.0%
Internal carotid artery	71	12.2%
Anterior cerebral artery	212	36.4%
Posterior circulation	170	29.3%
Treatment modality (clipping)	203	35.6%
Aneurysm irregularity	261	46.1%
Aneurysm daughter-sack	120	21.2%
Aneurysm sack size	6.6mm	4.0mm
Systemic parameters at admission		
WBC count (mean/SD)	11890/µl	4010/µl
CRP (mean/SD)	1.07 mg/dl	1.93 mg/dl
Troponin (mean/SD)	8.14 µg/l	66.03 µg/l
Maximum systolic blood pressure (mean/SD)	165	23
Maximum temperature (mean/SD)	37.2	0.8
Complications during SAH		
ICP increase requiring treatment	179	31.3%
Symptomatic angiographic vasospasm	138	23.7%
Systemic infections	168	32.2%
Outcome of SAH		
Cerebral infarction	202	35.1%
In-hospital mortality	47	8.1%
Unfavorable outcome at 6 months (mRS>3)	96	17.6%

*NSAID* non-steroidal anti-inflammatory drug, *ACE* angiotensin-converting enzyme, *AT1* angiotensin1, *WBC* white blood cells (count), *CRP* C-reactive protein

*Percentages were calculated according to the number of cases with known values

### Cerebral infarction

Of 576 aSAH individuals with at least one follow-up CT scan, cerebral infarcts were documented in 202 cases (35.1%), with no significant change (*p*=0.544) during the study time: 37.1%, 32%, and 34.9% for the periods 2003–2007, 2008–2012, and 2013–2016 respectively (hereinafter). Univariate analysis (Table 2) revealed a significant correlation between patients’ age, presence of arterial hypertension, use of calcium channel blockers (CCB) as regular medication (concomitant to standard nimodipine treatment), Fisher grades III–IV, presence of acute hydrocephalus, aneurysm clipping, and admission CRP >1.0 mg/dL with cerebral infarction. In the final multivariate analysis (Table 3), only CCB use (adjusted odds ratio [aOR]=2.63, *p*=0.002), acute hydrocephalus (aOR=2.33, *p*=<0.0001), and aneurysm clipping (aOR=1.78, *p*=0.003) were independently associated with the occurrence of cerebral infarction.

**Table 2:** Univariate analysis for the association between different baseline characteristics and primary study endpoints. For significant continuous and categorical variables, the test results after dichotomization were shown

Parameter	Cerebral infarction		In-hospital mortality		Unfavorable outcome at 6 mo.	
	OR (95% CI)/mean (SD)	*p*-value	OR (95% CI)/mean (SD)	*p*-value	OR (95% CI)/mean (SD)	*p*-value
Demographic characteristics						
Age>55 years	**1.70 (1.20–2.41)**	**0.003**	**4.49 (2.31–8.71)**	**<0.0001**	**3.51 (2.22–5.55)**	**<0.0001**
Sex (female)	1.23 (0.85–1.79)	0.304	1.62 (0.80–3.26)	0.196	0.88 (0.55–1.40)	0.630
Comorbidities						
Arterial hypertension	**1.75 (1.19**–**2.58)**	**0.005**	1.38 (0.70–2.72)	0.415	**2.06 (1.22**–**3.51)**	**0.006**
Hypothyroidism	0.70 (0.39–1.24)	0.266	0.74 (0.26–2.12)	0.807	0.68 (0.31–1.47)	0.377
Hyperthyroidism	0.65 (0.61–0.69)	0.303	0.92 (0.90–0.94)	1.000	0.82 (0.79–0.86)	0.592
Hyperuricemia	1.31 (0.49–3.50)	0.611	1.56 (0.35–7.06)	0.638	2.22 (0.75–6.54)	0.173
Diabetes mellitus	0.92(0.41–2.09)	1.000	0.39 (0.05–2.95)	0.500	0.93 (0.31–2.77)	1.000
Regular medication						
Statin	1.40 (0.65–3.03)	0.419	2.04 (0.67–6.15)	0.266	2.30 (0.96–5.51)	0.063
NSAID	0.92 (0.49–1.76)	0.872	1.46 (0.55–3.89)	0.399	1.06 (0.48–2.35)	0.838
Beta blockers	1.37 (0.84–2.23)	0.248	1.11 (0.48–2.57)	0.825	1.65 (0.92–2.97)	0.099
Calcium channel blockers	**3.32 (1.84**–**5.97)**	**<0.0001**	1.50 (0.60–3.71)	0.425	**2.19 (1.14**–**4.19)**	**0.030**
ACE inhibitors	1.11 (0.69–1.81)	0.709	1.22 (0.55–2.72)	0.665	1.63 (0.92–2.89)	0.108
AT1 receptor blockers	1.44 (0.68–3.02)	0.334	0.83 (0.19–3.58)	1.000	0.84 (0.28–2.50)	1.000
Anticoagulants	1.31 (0.70–2.45)	0.414	1.91 (0.76–4.79)	0.154	**2.44 (1.21**–**4.92)**	**0.017**
SAH characteristics						
Acute hydrocephalus	**2.64 (1.82**–**3.81)**	**<0.0001**	**4.03 (1.85**–**8.78)**	**<0.0001**	**3.93 (2.30**–**6.70)**	**<0.0001**
Fisher scale, grades III–IV	**1.58 (1.01**–**2.49)**	**0.047**	**4.95 (1.50**–**16.29)**	**0.003**	**3.81 (1.84**–**7.86)**	**<0.0001**
Aneurysm size	6.6 (±3.8) vs 6.5(±4.2)	0.691	6.6 (±4.0) vs 6.4 (±4.1)	0.506	6.5 (±4.0) vs 6.5 (±4.4)	0.658
Aneurysm irregularity	1.37 (0.74–2.52)	0.351	1.38 (0.97–1.95)	0.077	1.35 (086–2.12)	0.206
Daughter sack	1.35 (0.89–2.05)	0.162	1.57 (0.80–3.10)	0.187	1.48 (0.88–2.50)	0.155
Aneurysm location (posterior circulation)	0.97 (0.67–1.42)	0.924	0.82 (0.41–1.61)	0.619	0.79 (0.48–1.31)	0.364
Treatment modality (clipping)	**1.91 (1.34**–**2.73)**	**<0.0001**	**3.02 (1.60**–**5.72)**	**0.001**	**3.36 (2.11**–**5.34)**	**<0.0001**
Systemic parameters						
WBC count, 10^9^/L	11.77 (±4.12) vs 12.37 (±3.75)	0.093	11.89 (±3.97) vs 12.98 (±4.37)	0.083	11.88 (±4.03) vs 12.44 (±4.13)	0.238
CRP, mg/dL	1.01 (±1.97) vs 1.20 (±1.86)	0.296	1.04 (±1.86) vs 1.48 (±2.63)	0.156	**0.96 (±1.61) vs 1.36 (±2.15)**	**0.044**
Hemoglobin, g/dL	12.79 (±1.86) vs 12.64 (±2.00)	0.380	12.74(±1.87) vs 12.68 (±2.32)	0.941	12.74 (±1.82) vs 12.65 (±2.14)	0.844
Troponin, µg/L	9.79 (±80.60) vs 5.35 (±24.49)	0.998	8.7 (±68.85) vs 1.78 (±5.02)	0.254	7.59 (±68.46) vs 0.99 (±3.62)	0.232
Maximum systolic blood pressure	163 (±21) vs 168 (±25)	0.118	164 (±22) vs 169 (±28)	0.316	164 (±22) vs 169 (±28)	0.148
Maximum body temperature	37.2 (±0.7) vs 37.2 (±1.1)	0.658	37.2(±0.8) vs 37.2 (±0.7)	0.576	37.2 (±0.8) vs 37.1 (±0.8)	0.078

*OR* odds ratio, *SD* standard deviation, *NSAID* non-steroidal anti-inflammatory drug, *ACE* angiotensin-converting enzyme, *AT1* angiotensin1, *WBC* white blood cells (count), *CRP* C-reactive protein, *p*<0.05

**Table 3:** Multivariable analysis for the predictors of the primary study endpoints

Parameter	aOR (95% CI)	*p*-value
Cerebral infarction		
Age >55 years	1.44 (0.99–2.09)	0.057
Arterial hypertension	1.32 (0.86–2.01)	0.200
Calcium channel blockers	**2.63(1.42**–**4.86)**	**0.002**
Fisher scale, grades III–IV	1.06 (0.65–1.71)	0.822
Acute hydrocephalus	**2.33 (1.53**–**3.55)**	**<0.0001**
Treatment modality (clipping)	**1.78 (1.22**–**2.59)**	**0.003**
Treatment periods (5-years intervals)	0.85 (0.67–1.07)	0.169
In-hospital mortality		
Age >55 years	**4.24 (2.13**–**8.44)**	**<0.0001**
Fisher scale, grades III–IV	3.04 (0.92–10.07)	0.069
Acute hydrocephalus	**2.43 (1.06**–**5.58)**	**0.036**
Treatment modality (clipping)	**2.86 (1.50**–**5.46)**	**0.001**
Treatment periods (5-year intervals)	0.70 (0.46–1.06)	0.089
Unfavorable outcome at 6 months (mRS>3)		
Age >55 years	**2.77 (1.63–4.69)**	**<0.0001**
Arterial hypertension	1.54 (0.83–2.86)	0.166
Calcium channel blockers	1.28 (0.59–2.75)	0.527
Anticoagulants	2.10 (0.90–4.89)	0.086
Fisher scale, grades III–IV	**2.81 (1.22**–**6.49)**	**0.016**
Acute hydrocephalus	**2.22 (1.19**–**4.13)**	**0.012**
Treatment modality (clipping)	**3.98 (2.38**–**6.63)**	**<0.0001**
CRP >1.0 mg/dL at admission	**1.76 (1.04**–**2.96)**	**0.035**
Treatment periods (5-year intervals)	**0.64 (0.46**–**0.88)**	**0.006**

*aOR* adjusted odds ratio, *SD* standard deviation, *CRP* C-reactive protein, *p*<0.05

### In-hospital mortality

Forty-seven patients (8.1%) did not survive the initial aSAH treatment. The changes in the mortality rate over the study period (10.1% > 5.8% > 6.8%) did not reach statistical significance (*p*=0.218). Baseline characteristics associated with in-hospital mortality in univariate analysis (Table 2) were age, acute hydrocephalus, Fisher grades III–IV, and treatment modality. Finally, independent associations with mortality risk were confirmed in the multivariate analysis (Table 3) for higher age (>55 years, aOR=4.24, *p*=<0.0001), acute hydrocephalus (aOR=2.43, *p*=0.036), and aneurysm clipping (aOR=2.86, *p*=0.001).

### Unfavorable outcome at 6 months after SAH

The clinical follow-up data at 6 months after aSAH were available in 544 (93.5%) individuals. Of them, in 96 patients (17.6%), unfavorable outcome was documented. Interestingly, there was a trend towards decrease of the rate of unfavorable outcome in the analyzed 5-year intervals: 21.5% > 15.4% > 12.8% (*p*=0.076). Univariate analysis (Table 2) revealed a significant correlation between unfavorable outcome and age, arterial hypertension, regular medication with CCB and anticoagulants, Fisher grades III–IV, aneurysm clipping, and admission CRP. In the multivariable analysis (Table 3), age older than 55 years (aOR=2.77, *p*=<0.0001), acute hydrocephalus (aOR=2.22, *p*=0.012), Fisher grades III–IV (aOR=2.81, *p*=0.016), clipping (aOR=3.98, *p*<0.0001), and CRP>1.0 mg/dL (aOR=1.76, *p*=0.035) and treatment intervals (aOR=0.64 per-5-year-intervals, *p*=0.006) were independently related to poor 6-month functional outcome after aSAH.

**Fig. 1:**
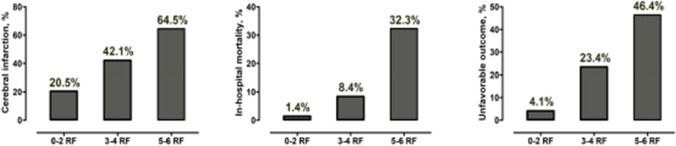
Rates of cerebral infarction, in-hospital mortality, and unfavorable outcome at 6 months (modified Rankin scale>3) depending on the number of present baseline risk factors (RF): age>55 years, acute hydrocephalus, Fisher grades III–IV, aneurysm clipping, calcium channel blocker medication (concomitant to nimodipine), and c-reactive protein > 1.0 mg/dL

Figure 1 visualizes the cumulative effect of all outcome-relevant baseline risk factors (age>55 years, acute hydrocephalus, Fisher grades III–IV, aneurysm clipping, CCB medication concomitant to nimodipine, and CRP> 1.0 mg/dL) which showed independent associations with at least one of the abovementioned primary study endpoints. The more baseline risk factors were present, the higher the burden of cerebral infarctions, in-hospital mortality, and unfavorable outcome at 6 months after aSAH.

### Complications during SAH

In 179 patients (31.3%), there was at least one ICP increase. Univariate analysis (Supplementary Table S1) revealed a significant correlation between ICP increase requiring treatment and arterial hypertension, acute hydrocephalus, Fisher grades III–IV, and aneurysm clipping. Moreover, there was a significant decrease of the rate of ICP increase over the study period (41.4 % > 31.8% > 8.7%, *p*<0.0001). In the final multivariate analysis (Supplementary Table S2), acute hydrocephalus (aOR=3.92, *p*<0.0001), aneurysm clipping (aOR=11.1, *p*<0.0001), and treatment periods (aOR=0.32 per-5-year-interals, *p*<0.0001) were independently associated with pathological ICP increase.

Intra-arterial spasmolysis for cerebral vasospasm was performed in 138 patients (23.7%). During the observation period, there was an increase in the number of cases with invasive vasospasm treatment: 18.1% > 30.6% > 26.5% (*p*=0.007). Baseline characteristics associated with this complication in univariate analysis (Supplementary Table S1) were age > 55 years, acute hydrocephalus, and higher Fisher grades III–IV. Independent associations with cerebral vasospasm treated with intra-arterial spasmolysis were confirmed in the multivariate analysis (Supplementary Table S2) with patients’ age (lower risk in individuals > 55 years, aOR=0.47, *p*=0.001), Fisher grades III–IV (aOR=2.21, *p*=0.028), CRP >1.0mg/dL (aOR=1.76, *p*=0.015), and treatment intervals (aOR=1.4, *p*=0.008).

Systemic infections were documented in 168 patients (32.2%). A slight decrease of the infections rate was observed during the study (34.6% > 32.3% > 27.7%, *p*=0.399). Univariate analysis (Supplementary Table S1) revealed a significant correlation for systemic infections with age >55 years, statin and CCB intake, acute hydrocephalus, and Fisher grades III–IV. In the final multivariate analysis (Supplementary Table S2), Fisher grades III–IV (aOR=2.38, *p*=0.004), acute hydrocephalus (aOR=2.19, *p*<0.0001), and aneurysm clipping (aOR=1.70,* p*=0.012) were independently associated with systemic infections.

**Fig. 2:**
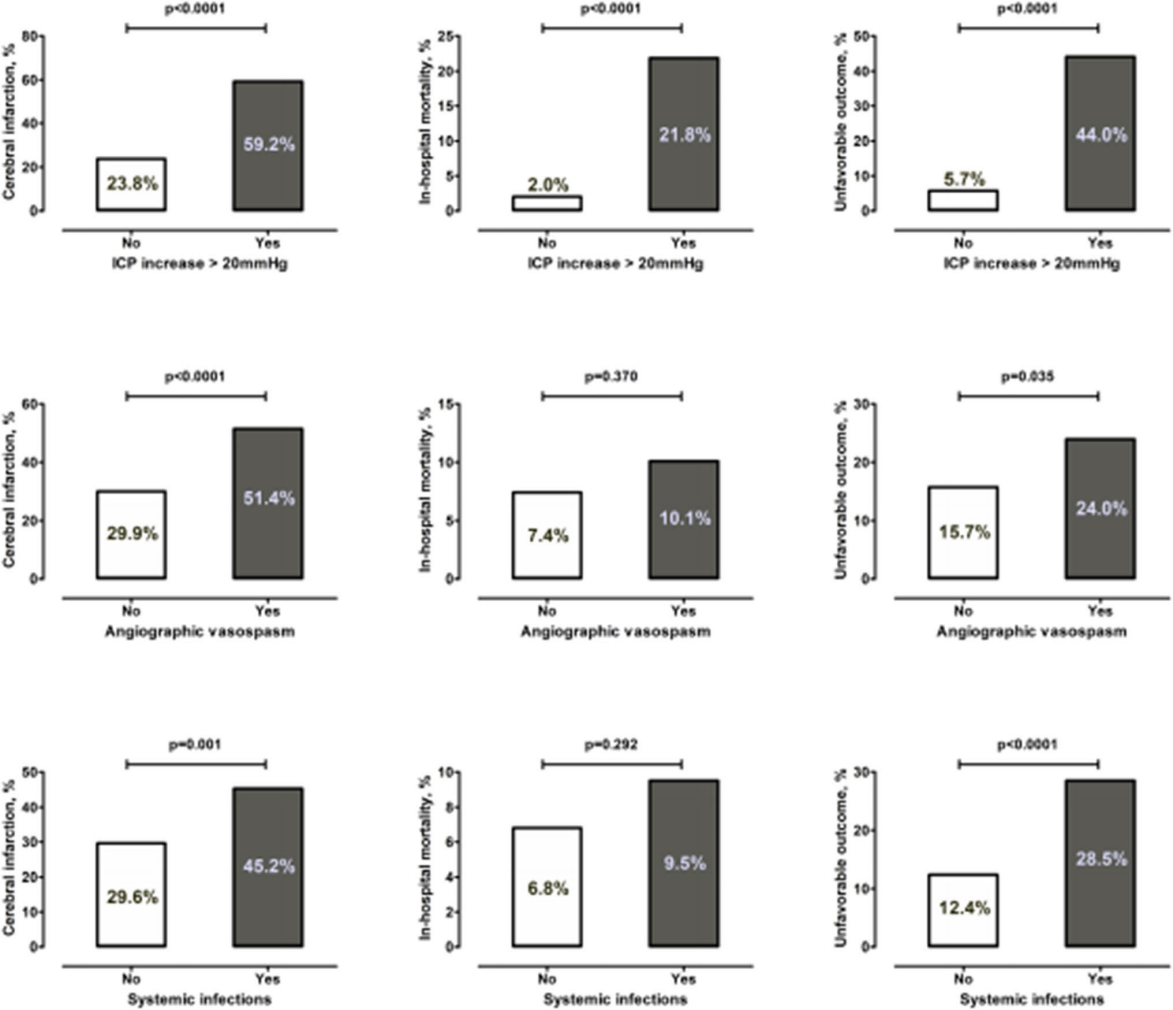
Impact of SAH complications (ICP increase >20mmHg, angiographic vasospasm, and systemic infections) on the risk of cerebral infarction, in-hospital mortality, and unfavorable outcome at 6 months (modified Rankin scale>3)

Figure 2 shows the association between the primary and secondary study endpoints. Patients with ICP increase>20 mmHg, angiographic vasospasm, and systemic infections showed higher rates of cerebral infarctions, in-hospital mortality, and 6-month unfavorable outcome.

## Discussion

The research question of our study aimed to identify risk factors in aSAH patients who faced poor outcome despite favorable initial clinical condition. We found a considerable rate of cerebral infarction in these aSAH individuals, but a relatively low burden of mortality and long-term morbidity. Several patient- and aSAH-related baseline characteristics were strongly associated with complications and poor outcome in aSAH cases with initial WFNS grades I–III.

For the estimation of outcome after aSAH, the grading of patients’ initial clinical condition based on the Hunt and Hess [17] or WFNS scales [31] is of high relevance [2, 12, 18, 32]. This strong link with aSAH outcome might be related to the fact that these clinical scales probably reflect the burden of EBI [30] after aneurysm rupture. In turn, EBI is considered the main cause of mortality and morbidity after aSAH [1, 3].

Patients with a lower grade of WFNS have a much better prognosis than patients with a higher grade, but even these patients may have a poor outcome despite a lower extent of EBI and maximal treatment [34]. In these patients, the effects of secondary complications like DCI, ICP increase, or infections might be of particular importance requiring measures for timely recognition and prevention of such adverse events. Moreover, the baseline parameters associated with a worse outcome of favorable-grade aSAH individuals require detailed analysis and inclusion in the assessment scores for early outcome prognostication and risk-adapted clinical management of aSAH patients after aneurysm securing [22].

To date, only few studies focused on the risk factors for complications and poor outcome in WFNS grade I–III aSAH patients. So, patients’ age, thick aSAH, obesity, and preadmission hypertension were revealed as risk factors for DCI and DCI-related poor outcome in favorable-grade aSAH in a multinational pooled cohort analysis [27]. In a post hoc analysis of the CONSCIOUS-1 trail data, 20% of aSAH patients presenting with WFNS grades I–II suffered from unfavorable outcome [9]. High admission systolic blood pressure, female sex, DCI, hyperthermia, respiratory system complications, and ICH were identified as independent predictors of poor outcome in this cohort. Moreover, in a study by Leira et al., subtle neurological deficits at baseline, which are not covered with the WFNS grading system, were associated with a worse outcome after 3 months in a favorable-grade aSAH cohort [22].

In our large retrospective monocentric study of WFNS grade I–III aSAH individuals, 35.1% of patients developed cerebral infarction(s) during disease, 8.1% died within the hospital stay, and 17.6% faced an unfavorable long-term outcome. In line with previous publications [4, 15, 26], we could confirm independent predictive value of patients’ age for disease morbidity and mortality in WFNS I–III-graded aSAH patients. The presence of acute hydrocephalus was also predictive for primary and secondary endpoints in our study. Fittingly, Benes et al. found that, in addition to patients’ age, hydrocephalus was a significant negative factor for outcome in patients admitted in favorable grade aSAH [4]. Due to pathologic ICP increase resulting in compromised cerebral perfusion, acute hydrocephalus might contribute to delayed neurological deterioration and poor outcome of initially well-performing aSAH patients. Therefore, early ICP monitoring and treatment are essential for the prevention of negative impact of acute hydrocephalus on the outcome of aSAH patients presenting with WFNS grades I–III.

Interestingly, aneurysm clipping was also independently associated with poor outcome and higher complication rates in our cohort of favorable-graded aSAH patients. The strongest clinical evidence on the possible impact of treatment modality on aSAH outcome arises from two large prospective trials—International subarachnoid aneurysm trial (ISAT) [24, 25] and Barrow Ruptured Aneurysm Trials (BRAT) [23]. Both trials showed the advantage of endovascular over microsurgical aneurysm treatment with regard to functional outcome. Although these trials were primarily not restricted to specific WFNS subgroups, the patients included in ISAT were mostly of favorable initial clinical condition [8]. In summary, there is a distinct negative impact of aneurysm clipping on functional outcome in initially favorable-graded aSAH patients. Of note, due to the presence of selection bias with microsurgical treatment of aneurysms not eligible for endovascular intervention, poorer treatment results after clipping in the cohorts treated in the post-ISAT-era might also be related to more complex aneurysms undergoing microsurgical treatment.

Then, CRP>1.0 mg/dL on admission as an inflammation marker showed independent association with occurrence of symptomatic cerebral vasospasm and unfavorable 6-month outcome. The severity of inflammatory response after aneurysm rupture is of high relevance for further course of disease impacting the probability of secondary complications and poor outcome [15]. Our data support the hypothesis on the essential role of inflammation for outcome of aSAH and underline the importance of the currently ongoing multicentric prospective trial evaluating the value of early anti-inflammatory therapy with glucocorticoids in aSAH patients [16].

Moreover, our analysis showed independent association between the use of CCB from previous medication (concomitant to standard nimodipine treatment) with the risk of cerebral infarction. As our study is based on a retrospective analysis of observational cohort, no judgment on eventual causal relationship between the medication use and occurrence of this complication is currently possible. However, several assumptions can be made requiring further evaluation and confirmation. In particular, concomitant cardiovascular comorbidity (as the indication for CCB prescription) might result in poorer cardiac output and therefore negatively impact cerebral perfusion after aSAH. Moreover, pharmacologic interactions between different drug classes might also play an essential role in the risk of development of cerebral infarction after aSAH. Further clinical and experimental research on different factors additionally impacting the outcome of favorable-graded aSAH patients is mandatory.

Of the remaining significant study findings, the improvement of the functional outcome and lower complications burden in favorable-graded aSAH patients over the study period should be particularly emphasized. Although the changes in the infraction and in-hospital mortality rates did not reach statistical significance, a nearly 2-fold decrease of the rate of unfavorable 6-month outcome between the initial and the last study periods (21.5% > 12.8%) was observed. Due to retrospective and observational nature of the present study, no direct causal conclusions on the backgrounds of this functional improvement can be made upon our data. However, lower rate of pathologic ICP increase, more frequent utilization of intra-arterial spasmolysis for vasospasm treatment, and somewhat decreasing rate of systemic infections in the cohort during the study period might have contributed to better final outcome of our aSAH patients. As possible explanation for this lower burden of ICP and infection-related complications, changes in the conservative SAH management [3], and particularly the gradual reduction in the practice of using triple H therapy [14] should be mentioned. Fittingly, there was more frequent use of intra-arterial spasmolysis for vasospasm treatment in the investigated cohort during this time. For more detailed analysis of the effect of changes of treatment regimens on SAH course and outcome, further studies are mandatory.

### Limitations

As already mentioned, the major limitation of our study is its monocentric and retrospective observational design, with all related information and selection bias. Therefore, the findings of our study require further confirmation in external observational aSAH cohorts and in the context of prospective clinical trials. Nevertheless, our analysis is based on one of the largest series of patients with aSAH and may provide insights for further research aimed at optimizing treatment approaches for those patients with initial favorable WFNS grade.

## Conclusion

Although cerebral infarction is a common complication in aSAH individuals with favorable initial clinical condition, >90% of these patients survive the initial treatment and show favorable long-term outcome in >80% of cases. SAH patients in advanced age, with high burden of intracranial bleeding, acute hydrocephalus, aneurysm clipping, early systemic inflammatory response, and concomitant CCB medication, are of particular risk for secondary complications and poor outcome despite favorable initial clinical condition. Our findings could help to optimize treatment strategies and prevent complications in aSAH patients who tolerated well the initial bleeding event.

## Supplementary information

Below is the link to the electronic supplementary material.

Supplementary file1 (DOCX 24 KB)

## Data Availability

The data presented in this study are available on request from the corresponding author. The data are not publicly available due to privacy.

## Abbreviations

aSAH: Aneurysmal subarachnoid hemorrhage
WFNS: World Federation of Neurosurgical Societies
EBI: Early brain injury
ICH: Intracerebral hemorrhage
IVH: Intraventricular hemorrhage
DCI: Delayed cerebral ischemia
CT: Computed tomography
DSA: Digital subtraction angiography
EVD: External ventricular drain
ICP: Intracranial pressure
TCD: Transcranial Doppler ultrasound sonography
WBC: White blood cells
CRP: C-reactive protein
mRS: Modified Rankin Scale
NSAID: Non-steroidal anti-inflammatory drug
ACE: Angiotensin-converting enzyme
AT1: Angiotensin1
CCB: Calcium channel blockers
OR: Odds ratio
CI: Confidence interval
RF: Risk factors
ISAT: International Subarachnoid Aneurysm Trial
BRAT: Barrow Ruptured Aneurysm Trials
