# Supplementary material for: Risk factors for poor outcome after aneurysmal subarachnoid hemorrhage in patients with initial favorable neurological status
Source: Acta Neurochir (Wien). 2024 Feb 20;166(1):93. doi: 10.1007/s00701-024-05968-5 (PMC10879324; doi:10.1007/s00701-024-05968-5)
Supplement: Supplementary file 1 — Supplementary file1 (DOCX 24 KB) [file 701_2024_5968_MOESM1_ESM.docx]

**ONLINE SUPPLEMENTS**

*Supplementary Table S1: Univariate analysis for the association between previous medical history, SAH characteristics and complications of SAH*

| Parameter | ICP increase | | Symptomatic vasospasm | | Systemic infection | |
| --- | --- | --- | --- | --- | --- | --- |
|  | **OR (95% CI) / mean (SD)** | **p-value** | **OR (95% CI) / mean (SD)** | **p-value** | **OR (95% CI) / mean (SD)** | **p-value** |
| Age>55 years | 0.95 (0.66 -1.37) | 0.854 | **0.62 (0.41-0.93)** | **0.022** | **1.48 (1.02 -2.14)** | **0.044** |
| Sex (female) | 0.91 (0.63-1.33) | 0.700 | 1.34 (0.88 -2.05) | 0.177 | 0.69 (0.47 -1.02) | 0.071 |
| Arterial hypertension | **1.55 (1.04-2.31)** | **0.032** | 1.12 (0.74 -1.70) | 0.675 | 1.45 (0.96 -2.19) | 0.085 |
| Hypothyroidism | 0.76 (0.42 -1.38) | 0.465 | 1.08 (0.59 -1.97) | 0.758 | 0.83 (0.45 -1.52) | 0.650 |
| Hyperthyroidism | 0.68 (0.65 -0.72) | 0.332 | 0.76 (0.73 -0.80) | 0.597 | 0.68 (0.64 -0.72) | 0.311 |
| Hyperuricemia | 1.21(0.44 -3.32) | 0.791 | 0.20 (0.03 -1.51) | 0.140 | 2.18 (0.80 -5.90) | 0.171 |
| Diabetes mellitus | 0.46 (0.17 -1.23) | 0.144 | 1.23 (0.353 -2.85) | 0.655 | 0.81 (0.33 -1.98) | 0.827 |
| Statin | 1.29 (0.58 -2.88) | 0.530 | 1.85 (0.83 -4.12) | 0.168 | **2.58 (1.17 -5.71)** | **0.029** |
| NSAID | 0.76 (0.38 -1.51) | 0.509 | 1.48 (0.77 -2.87) | 0.275 | 0.85 (0.43 -1.66) | 0.739 |
| Beta blockers | 1.36 (0.83 -2.22) | 0.243 | 1.31 (0.77 -2.21) | 0.327 | 1.19 (0.71 -2.0) | 0.503 |
| Calcium channel blockers | 1.16 (0.64 -2.12) | 0.640 | 1.00 (0.52 -1.92) | 1.000 | **2.27 (1.23 -4.19)** | **0.012** |
| ACE inhibitors | 0.77 (0.46 -1.30) | 0.376 | 1.03 (0.60 -1.76) | 0.892 | 1.23 (0.75 -2.03) | 0.436 |
| AT1 receptor blockers | 1.15 (0.52 -2.52) | 0.838 | 0.63 (0.24 -1.68) | 0.508 | 1.12 (0.49 -2.56) | 0.831 |
| Anticoagulants | 1.15 (0.60 -2.20) | 0.735 | 0.59 (0.26 -1.35) | 0.269 | 1.28 (0.67 -2.44) | 0.497 |
| Acute hydrocephalus | **4.04 (2.69 -6.06)** | **<0.0001** | **1.85 (1.24 -2.77)** | **0.003** | **2.88 (1.93-4.31)** | **<0.0001** |
| Fisher scale, grade III-IV | **2.54 (1.52 -4.24)** | **<0.0001** | **2.61 (1.47 -4.63)** | **0.001** | **3.16 (1.82 -5.49)** | **<0.0001** |
| Aneurysm size | 6.6 (±3.7) vs  6.6 (±4.6) | 0.340 | 6.6 (±4.0) vs  6.5 (±3.9) | 0.782 | 6.5 (±3.6) vs  6.8 (±4.9) | 0.760 |
| Aneurysm location (posterior circulation) | 0.68 (0.46 – 1.02) | 0.074 | 0.67 (0.43 – 1.04) | 0.086 | 1.16 (0.77 – 1.73) | 0.533 |
| Aneurysm irregularity | 1.14 (0.80 -1.64) | 0.519 | 1.23 (0.83 -1.81) | 0.322 | 1.14 (0.79- 1.66) | 0.504 |
| Daughter sack | 0.94 (0.60 -1.46) | 0.823 | 1.37 (0.87 -2.16) | 0.184 | 0.73 (0.46 -1.18) | 0.203 |
| Treatment modality (clipping) | **7.34 (4.93 -10.92)** | **<0.0001** | 1.08 (0.73-1.61) | 0.759 | **1.82 (1.25 -2.66)** | **0.002** |

*ABBREVIATIONS: OR - odds ratio; SD – standard deviation; NSAID – non-steroidal anti-inflammatory drug; ACE – Angiotensin-converting enzyme; AT1 – Angiotensin1; WBC – white blood cells (count); CRP – C-reactive protein.*

*Supplementary Table S2: Multivariable analysis for predictors of SAH complications*

| Parameter | OR (95% CI) | p-value |
| --- | --- | --- |
| **ICP increase requiring treatment** | | |
| Arterial hypertension | **1.81 (1.10 –2.99)** | **0.020** |
| Fisher scale, grade=3-4 | 1.75 (0.91 –3.38) | 0.094 |
| Acute hydrocephalus | **3.92 (2.37 –6.49)** | **<0.0001** |
| Treatment modality (clipping) | **11.10 (6.83 –18.03)** | **<0.0001** |
| Treatment periods (5-years intervals) | **0.32 (0.23 –0.45)** | **<0.0001** |
| **Symptomatic angiographic vasospasm** | | |
| Age >55 years | **0.47 (0.31 –0.73)** | **0.001** |
| Fisher scale, grade=3-4 | **2.21 (1.09 –4.47)** | **0.028** |
| Acute hydrocephalus | 1.54 (0.98 –2.44) | 0.063 |
| CRP >1.0 mg/dL | **1.76 (1.11 –2.77)** | **0.015** |
| Treatment periods (5-years intervals) | **1.40 (1.09 –1.76)** | **0.008** |
| **Systemic infections** | | |
| Age >55 years | 1.24 (0.81 –1.90) | 0.364 |
| Calcium channel blockers | 1.71 (0.80 –3.68) | 0.163 |
| Statin | 2.12 (0.93 -4.86) | 0.075 |
| Fisher scale, grade=3-4 | **2.38 (1.34 –4.24)** | **0.004** |
| Acute hydrocephalus | **2.19 (1.41 –3.38)** | **<0.0001** |
| Treatment modality (clipping) | **1.70 (1.12 –2.57)** | **0.012** |
| Treatment periods (5-years intervals) | 0.79 (0.62 –1.00) | 0.051 |

*ABBREVIATIONS: OR - odds ratio; SD – standard deviation; CRP – C-reactive protein.*

**Risk factors for poor outcome after aneurysmal subarachnoid hemorrhage in patients with initial good neurological status**

**Acta Neurochirurgica**

Annika Lenkeit^1^, Marvin Darkwah Oppong^1^, Thiemo Florin Dinger^1^, Meltem Gümüs^1^, Laurèl Rauschenbach^1^, Mehdi Chihi^1^, Yahya Ahmadipour^1^, Anne-Kathrin Uerschels^1^, Philipp Dammann^1^, Cornelius Deuschl^2^, Karsten H. Wrede^1^, Ulrich Sure^1^, Ramazan Jabbarli^1^

¹ Department of Neurosurgery and Spine Surgery, University Hospital Essen, Essen, Germany

² Department of Diagnostic and Interventional Radiology and Neuroradiology, University Hospital Essen, Essen, Germany

Email: [annika.lenkeit@uk-essen.de](mailto:annika.lenkeit@uk-essen.de)
